# Magnetoactive, *Kirigami*-Inspired Hammocks to Probe Lung Epithelial Cell Function

**DOI:** 10.1007/s12195-024-00808-z

**Published:** 2024-07-08

**Authors:** Katherine Wei, Avinava Roy, Sonia Ejike, Madeline K. Eiken, Eleanor M. Plaster, Alan Shi, Max Shtein, Claudia Loebel

**Affiliations:** 1 Materials Science & Engineering, College of Engineering, University of Michigan, Ann Arbor, USA; 2 School of Dentistry, University of Michigan, Ann Arbor, USA; 3 Biomedical Engineering, College of Engineering, University of Michigan, Ann Arbor, USA

**Keywords:** Dynamic culture, Mechanotransduction, Lung epithelial cells, Magnetic actuation Kirigami

## Abstract

**Introduction:**

Mechanical forces provide critical biological signals to cells. Within the distal lung, tensile forces act across the basement membrane and epithelial cells atop. Stretching devices have supported studies of mechanical forces in distal lung epithelium to gain mechanistic insights into pulmonary diseases. However, the integration of curvature into devices applying mechanical forces onto lung epithelial cell monolayers has remained challenging. To address this, we developed a hammock-shaped platform that offers desired curvature and mechanical forces to lung epithelial monolayers.

**Methods:**

We developed hammocks using polyethylene terephthalate (PET)-based membranes and magnetic-particle modified silicone elastomer films within a 48-well plate that mimic the alveolar curvature and tensile forces during breathing. These hammocks were engineered and characterized for mechanical and cell-adhesive properties to facilitate cell culture. Using human small airway epithelial cells (SAECs), we measured monolayer formation and mechanosensing using F-Actin staining and immunofluorescence for cytokeratin to visualize intermediate filaments.

**Results:**

We demonstrate a multi-functional design that facilitates a range of curvatures along with the incorporation of magnetic elements for dynamic actuation to induce mechanical forces. Using this system, we then showed that SAECs remain viable, proliferate, and form an epithelial cell monolayer across the entire hammock. By further applying mechanical stimulation via magnetic actuation, we observed an increase in proliferation and strengthening of the cytoskeleton, suggesting an increase in mechanosensing.

**Conclusion:**

This hammock strategy provides an easily accessible and tunable cell culture platform for mimicking distal lung mechanical forces in vitro. We anticipate the promise of this culture platform for mechanistic studies, multi-modal stimulation, and drug or small molecule testing, extendable to other cell types and organ systems.

**Supplementary Information:**

The online version contains supplementary material available at 10.1007/s12195-024-00808-z.

## Introduction

**Fig. 1:**
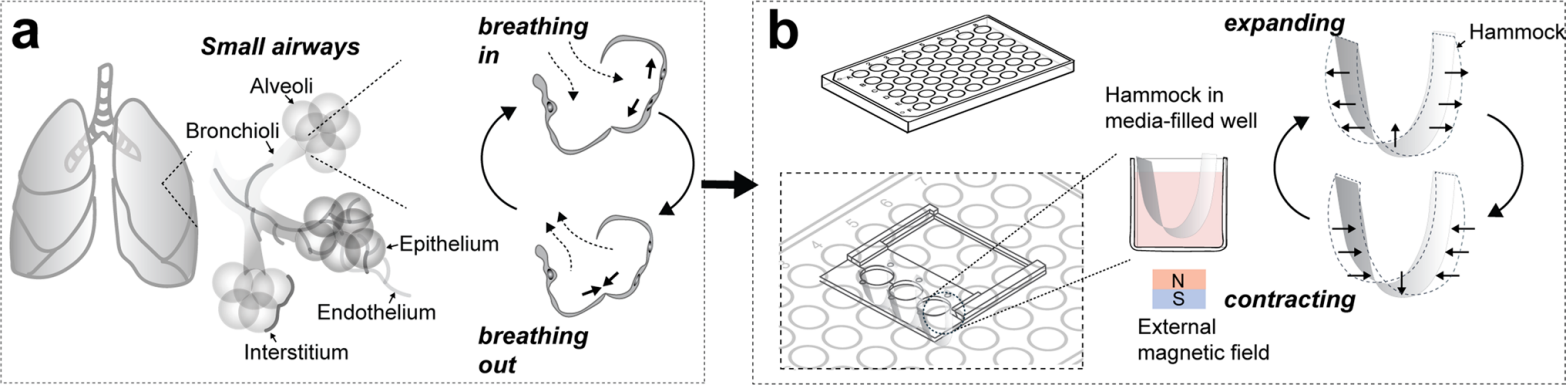
Engineered platform to mimic breathing movements in vitro. **a** Schematic illustrating the structure and architecture of the small airways that are under continuous expansion and contraction during breathing.** b** Schematic illustrating the engineered hammock-shaped platform with membranes of polyethylene terephthalate (PET) fixated onto a sliding hammock holder customized to fit into individual media-filled wells of a 48 well plate. Additional modification of the PET membranes with silicone elastomer including ferromagnetic neodymium–iron–boron (NdFeB) microparticles enables magnetic actuation to mimic the expansion and contraction of the small airways

The distal lung is composed of small airways and alveoli, the tiny sacs within the lung where the exchange of oxygen and carbon dioxide takes place. The rhythmic contraction and expansion of the respiratory muscles cause the necessary changes in pulmonary pressure, driving air exchange to achieve pulmonary ventilation [1]. The resulting mechanical forces act on various cells within the distal airway, including mesenchymal, endothelial, and epithelial cells, which are necessary for maintaining the function and structural integrity of the distal airways [2]. During normal breathing, the distal airway epithelium undergoes periodic mechanical strain (~ 5 to 12%) at each respiratory cycle (Fig. 1a) [3]. A disruption of physiological mechanical forces has been shown to contribute to the onset and progression of diseases such as pulmonary fibrosis, chronic obstructive pulmonary disease, and emphysema [4, 5], indicating that the distal airways present a complex mechanical environment that impacts cell proliferation, morphology, and function [4, 6].

Multiple approaches and tools have been developed to explore the mechanisms of mechanotransduction in lung cells [1, 7, 8], including uniaxial or biaxial cell stretch stimulation [9–12], cyclic pressure devices [13–15], and microfluidic organ-on-chip devices [16, 17]. Additionally, the architecture of the underlying extracellular matrix (ECM), including curvature, plays an essential role in directing cellular organization, function and responses to environmental stimuli [5, 18]. Therefore, to recreate the unique alveolar architecture and dynamic mechanical environment, in vitro models need to incorporate both curvature and mechanical stimulation. Previous work introduced a microfluidic device based on stretchable membranes that deflect into curved-like structures upon applying a vacuum [13]. Another recent approach used embedded sacrificial microbeads into 3D hydrogels to fabricate 3D, stretchable alveolar shapes within a microfluidic device [19]. However, while stretchable membranes and 3D alveolar structures represent a significant advancement, studies of mechanosignaling and transduction are often challenging in 3D hydrogels, and configuring and operating microfluidic devices may be inconvenient for a biology lab workflow and skillset.

Here, we report on a hammock-shaped platform, inspired by the Japanese paper art of *kirigami*, that facilitates the culturing of cells on curved substrates within common multi-well cell culture plates to recapitulate the curvature and mechanical forces within the distal lung. The hammocks are made by laser cutting thin polyethylene terephthalate (PET) membranes and mounting them on 3D printed holders. The dimensions are tuned such that an individual hammock fits into an individual well within 48-well plates and are shown to be easily customizable to integrate with other well plate designs. The hammock holders are designed to hold several hammocks sitting in adjacent wells. Building upon recent work with programmable elastomers [20–23], our platform further incorporates magnetoactive silicone elastomer membranes integrated with the PET membranes to provide remote dynamic mechanical stimulation upon applying a magnetic field (Fig. 1b).

## Methods

### PET Membrane and Hammock Holder Fabrication and Characterization

PET membranes for hammock-shaped platform were laser cut from a 23 µm thick PET membrane (Goodfellow) into 2D patterns using a Universal Laser System laser cutter. The cut parameters were set at 2% power, 6.5% speed, and 1000 PPI using a 1.5 optic. The hammock holder was made of two 3D printed components fabricated using an Original Prusa i3 MK3 printer and PET glycol filaments. The printing parameters were set with a 0.15 mm layer height, 15% infill, and support materials which were removed after printing. Local curvatures of the hammocks, defined as the inverse of the radius of curvature of circles tangential to points along the hammock were computed using the ImageJ Kappa-Curvature Analysis plugin. For each hammock, the shape was traced with a B-Spline curve, followed by addition of Kappa created data points and curvature values based on the fitted curve. Obtained data points were used to create scatter plots followed by assigned curvature heat maps using OriginPro software.

### Cell Seeding and Culture

Human lung fibroblasts (up to passage 3) were expanded in Dulbecco’s modified Eagle medium (DMEM) with 10% fetal bovine serum, followed by trypsinizing in 0.05% Trypsin/EDTA for 5 min at 37 °C. Flat PET membranes were sterilized within a germicidal ultraviolet light box followed by seeding single cell suspensions atop with 80 µL media per membrane at a density of 15 × 10^3^/cm^2^. Human umbilical vein endothelial cells (up to passage 6) were expanded in Endothelial cell growth medium (EGM-2, Lonza), followed by trypsinizing in 0.05% Trypsin/EDTA for 5 min at 37 °C and seeded at a density of 50 × 10^3^/cm^2^ PET membrane resuspended in 80 µL media per membrane. Human bronchial epithelial cells (up to passage 3) were expanded in bronchial epithelial cell growth medium (BEGM, Lonza), trypsinized with 0.25% Trypsin/EDTA for 5 min at 37 °C and seeded at a density of 40 × 10^3^/cm^2^ PET membrane resuspended in 80 µL media per membrane. Human small airway epithelial cells (SAECs) (up to passage 3) were expanded in small airway epithelial cell media (SAGM, Lonza), trypsinized with 0.25% Trypsin/EDTA for 5 min at 37 °C and seeded at a density of 30–50 × 10^3^/cm^2^ PET membrane resuspended in 80 µL media per membrane. Cell suspensions on flat PET membranes were incubated either for 1 h (fibroblasts) or 2 h (endothelial and epithelial cells) at 37 °C prior submersion into media-filled single wells of a 48 well plate and cultured for 48 h at 37 °C. For flat controls, PET membrane were cut into 10 mm strips and cultured individually in media-filled single wells of a 24 well plate and cultured for 48 h at 37 °C. For dynamic culture, cell-seeded hammocks were placed atop an electromagnet within the incubators after an initial 24 h of static culture.

### Cell Staining, Immunofluorescence, and Quantification

Cell-laden samples were fixated in 4% paraformaldehyde for 30 min, incubated in 0.1% Triton-X for 20 min at 4 °C and blocked with 2 wt% bovine serum albumin for 1 h at room temperature. Primary antibodies (yes-associated protein/transcriptional coactivator with PDZ-binding motif (YAP/TAZ): Santa Cruz Technologies cat. no. sc-101199, 1:200; ZO-1: Cell Signaling cat. no. D6L1E, 1:400; Pan cytokeratin: Novus Bio cat. no. NBP2-29429, 1:200; Ki-67: Invitrogen eBioscience^TM^ cat. no. 14-5698-82, 1:200) were incubated overnight at 4 °C, followed by 1 h incubation in secondary antibody (Abcam cat. no. ab150113,1:200), phalloidin (Invitrogen cat. no. A22287, 1:200) and Hoechst (1:1000, cat. no. 62249, Thermo Scientific). All samples were imaged with a Leica THUNDER and F-Actin and pan-cytokeratin images were computationally cleared with the THUNDER software. The percentage of Ki67 positive cells was quantified by dividing the number of Ki67 positive cell nuclei by the total number of nuclei within each image. Cytokeratin intensities were quantified on the sum projections of ROIs of single cells of computationally cleared images using previously published protocols [24, 25].

### Magnetic PDMS (MagPDMS) and PET-MagPDMS Fabrication

MagPDMS membranes were fabricated by mixing 6 g NdFeB particles (5 µm, MagneQuench) with 4 g polydimethylsiloxane (PDMS, 9:1 ratio of base and crosslinker) to obtain 17% volume fraction of magnetic particles in PDMS. The mixture was carefully poured onto a Sigmacote-treated coverglass with a second Sigmacote-treated cover glass placed on top to obtain 100–200 µm thick films, followed by an overnight curing process in a pre-heated oven set at 75 °C. Upon curing, the MagPDMS film was removed from the coverglass, cut into 14 × 4 mm pieces and attached to the center of plasma-etched PET membranes. Upon a second round of plasma-etching, a second magPDMS film was attached to enhance magnetic actuation. Next, the NdFeB dipoles within the PET-MagPDMS membranes were aligned using a 1 T magnetic field followed by attachment to the hammock holders.

### Magnetic Actuation of PET-MagPDMS Constructs

The programmed PET-MagPDMS membranes were actuated with a magnetic field (0–120 mT) generated from an electromagnet (Bunting BDE-4032-12) operated with a DC power supply (Tekpower TP3005P). A Dino-Lite edge USB camera was used to record the actuation states, and the images were analyzed using ImageJ (version: 1.8). For cell-culture experiments, the electromagnet was integrated in a circuit controlled using an Arduino Mega 2560 micro-controller while an IRF520 N-channel MOSFET was used for switching the circuit on (65 mT)/ off (0 mT) and a frequency of 15 cycles per minute.

### Statistical Analyses and Reproducibility

Statistical analyses were performed using GraphPad Prism 10 software. For assessing differences between two distinct experimental conditions, two-tailed Student’s *t*-tests were applied, whereas comparisons involving multiple groups used one-way ANOVA followed by Bonferroni post hoc tests. No data were excluded from the analyses. All experimental procedures were replicated in no fewer than three independent experiments and as outlined in the manuscript.

## Results and Discussion

### Hammock-Shaped Platform Design and Characterization

**Fig. 2:**
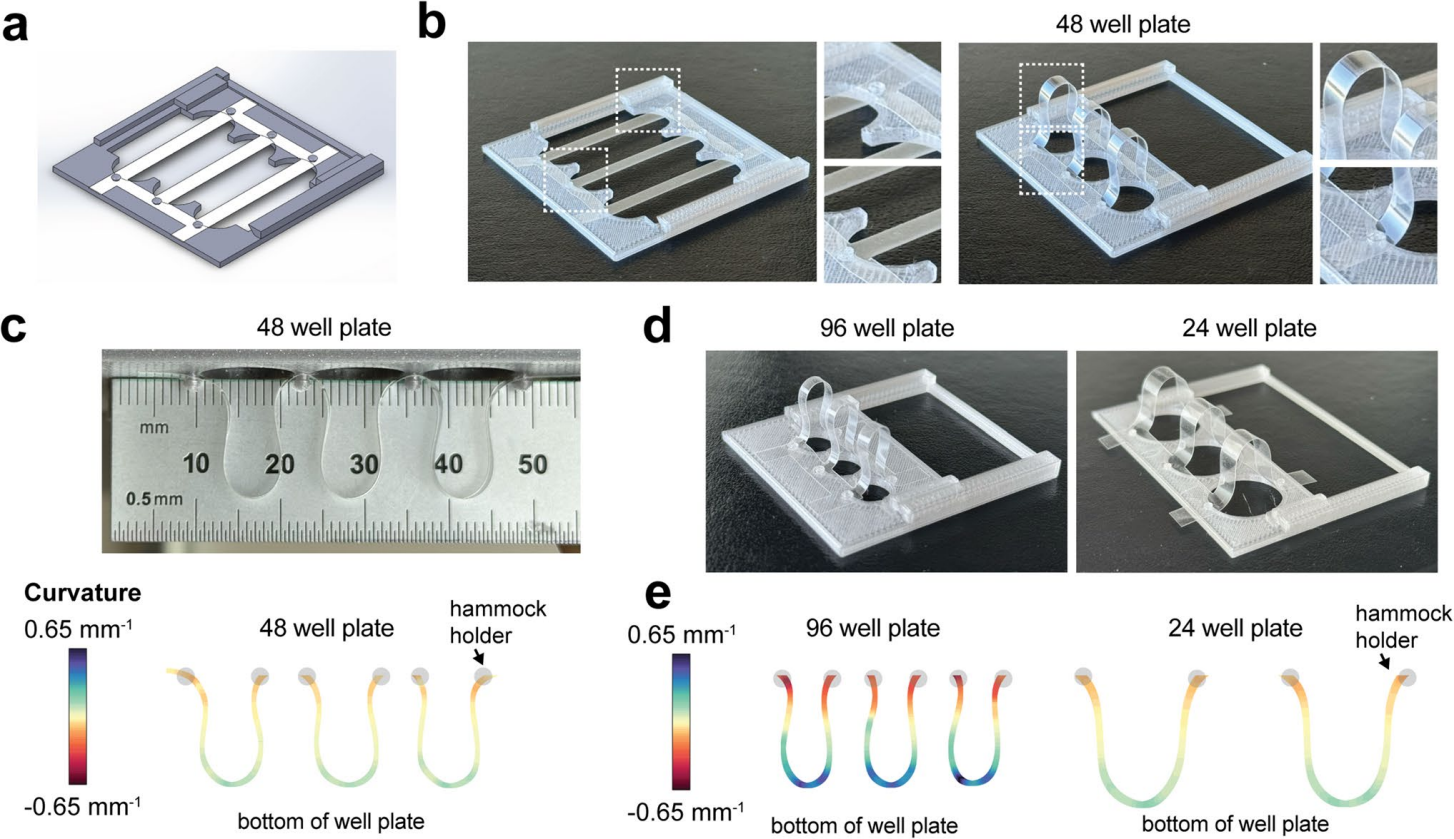
Hammock-shaped platform design and characterization. **a** Solidworks schematic (top) illustrating the printed holder extended to its entire length and flat PET hammocks secured to it with dimensions (bottom) that enable fit into a 48 well plate (dimensions in mm). Circles are 1.6 mm in diameter with three holes equally spaced along the perimeter that are each 0.3 mm long. **b** Representative photographs of the PET hammocks secured to the holder in extended flat position (left) and when curved upon sliding the holder back (right). **c** Representative photograph and curvature heatmap of hammocks for 48 well plates and calculated curvature. **d** Representative photographs of the PET hammocks secured to the holder for 96 and 24 well plates. **e** Curvature heatmaps of hammocks for 96 and 24 well plates

The scaffold consists of the PET hammock membrane and the 3D printed hammock holder designed in SolidWorks 3D modeling software. The holder is made of two pieces, an outer frame and a sliding insert. The laser-cut PET membranes contain “bridges” whose width is smaller than the diameter of each well (here, a conventional 48-well plate) (Fig. 2a). The length of each bridge is designed such that upon sliding the movable part of the holder, a curved-hammock shape is created (Fig. 2b), that is able to protrude down into adjacent wells (Supplementary Fig. 2). When placed into individual wells, the hammocks can be fully immersed into media. Towards engineering these hammocks to study cell mechanosensing, we next quantified the local curvature based on representative photographs. Due to the folding upon moving the sliding component, a positive curvature (convex) is generated at the membrane near the plane of the holder, whereas negative curvatures (concave) are generated at the bottom of the membrane (Fig. 2c). Both the membrane and holder dimensions were customized to also integrate with 96 and 24 well plate designs to either decrease or increase the radius of curvature (Fig. 2d). All hammocks show differences in curvature distribution as a function of the location on the hammocks which may be useful for studies of local mechano-signaling and responses in curved environments (Fig. 2e). It is important to note that the size and thus the curvatures of distal lung structures vary among species, including from ~ 30 to 100 µm from mice to pigs [26] and ~ 200 µm in humans [27] and ~ 300 µm in race horses [28]. Thus, we have created hammock culture scaffolds that are readily customizable by adjusting the length and spacing of the PET membranes, as well as the comprising materials.

### Small Airway Epithelial Cells Respond to Hammock Curvature

**Fig. 3:**
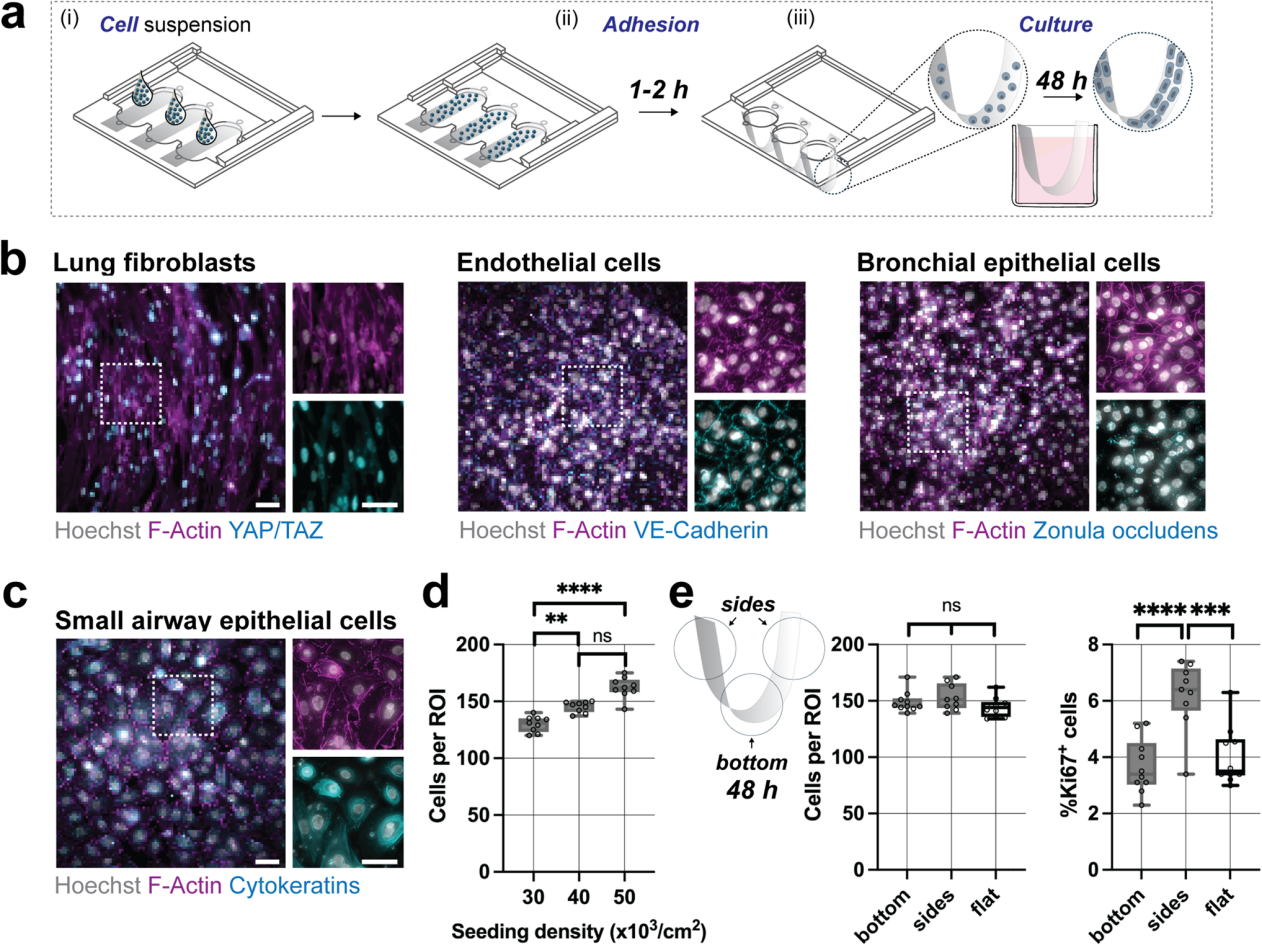
Culture of small airway cells on static hammock-shaped platform. **a** Schematic illustrating the cell seeding and culture protocol: (i) Single cells were seeded on top of the flat PET membranes and (ii) left for 1–2 h to settle and adhere to the substrate, followed by sliding the holder to form hammocks within each well. (iii) Cells were then cultured for 48 h before analysis. **b** Representative images of human lung fibroblasts [F-actin (magenta), YAP/TAZ (cyan)], human umbilical vein endothelial cells [F-actin (magenta), VE-Cadherin (cyan)], and human bronchial epithelial cells (F-actin (magenta), zonula occludens (cyan)) at 48 h in culture (scale bars 50 µm). **c** Representative image of human small airway epithelial cells (F-actin (magenta), pan-cytokeratin (cyan)) at 48 h in culture (scale bars 50 µm). **d** Quantification of the cell density per region of interest (ROI) at 48 h in culture, and from 3 independent experiments (**p < 0.01, ****p < 0.0001, ns = not significantly different by ANOVA and Bonferroni’s multiple comparisons test). **e** Schematic and quantification of local differences in cell density (cells per ROI) and proliferation (Ki67 positive cells per total cell number, see Supplementary Fig. 4 for representative images) at the bottom and sides of the hammocks and flat controls (***p < 0.0001, ns = not significantly different by Student’s *t*-test)

To assess the hammock system’s utility as a cell culture platform to study the effects of curvature, we first seeded cells with an average of 40 × 10^3^ cells per cm^2^ PET surface area in cell-type specific media. Upon initial adhesion on flat membranes, we next slid the hammock holder to have the flat PET membranes assume curved shapes and cultured cells for up to 48 h within the hammocks (Fig. 3a). To show the versatility of the system, we cultured fibroblasts, endothelial and epithelial cells which are all representative cell types of the distal airways. Within 48 h, all cells formed a continuous monolayer as indicated by F-Actin staining. Fibroblasts showed elongated cell morphology and high nuclear localization of YAP/TAZ, suggesting that the hammocks support cellular spreading. Both endothelial and epithelial cells showed the formation of strong cell-to-cell adhesions as indicated by the staining of markers for adherens junctions VE-Cadherin (endothelial junctions) and ZO-1 (zonula occludens, epithelial junctions) (Fig. 3b). These findings confirm that PET membranes support the adhesion and monolayer formation of mesenchymal, endothelial and epithelial cell types. Having shown that these cells adhere, we next used primary SAECs to assess how the mechanical properties of the hammocks regulate distal airway cell function. SAECs similarly formed a monolayer within 48 h of culture with cortical actin fibers and cytokeratin network throughout the cytoplasm indicating healthy SAEC function (Fig. 3c). Initial cell seeding density may direct monolayer formation. Thus, we seeded SAECs at average densities of 30 × 10^3^, 40 × 10^3^, and 50 × 10^3^ per cm^2^ PET and quantified cell numbers post-monolayer formation. After 48 h, a monolayer was observed for all conditions (Supplementary Fig. 4) whereas cell numbers did not further increase for seeding densities higher than 40 × 10^3^ per cm^2^ PET (Fig. 3d). One concern, when culturing cells on curved substrates, is the gravitational accumulation of cells preferring concave regions. Thus, we analyzed the number of cells at different locations across the hammocks and compared to flat PET membranes. No significant differences were observed in the number of cells on the bottom (± 5 mm from the center) versus the side (> 5 mm from the center) and flat controls, whereas cells on the sides showed a trend towards higher proliferation as shown by the increase in Ki67 positive cells (Fig. 3e, Supplementary Fig. 4). Taken together, these finding indicate that PET hammocks support the adhesion and monolayer formation of several cell types similar to flat PET membranes. In addition, SAECs preferentially proliferate at the sides of the hammocks, perhaps due to the least negative curvature (Fig. 2c).

### Design of Magneto-Active Hammocks

**Fig. 4:**
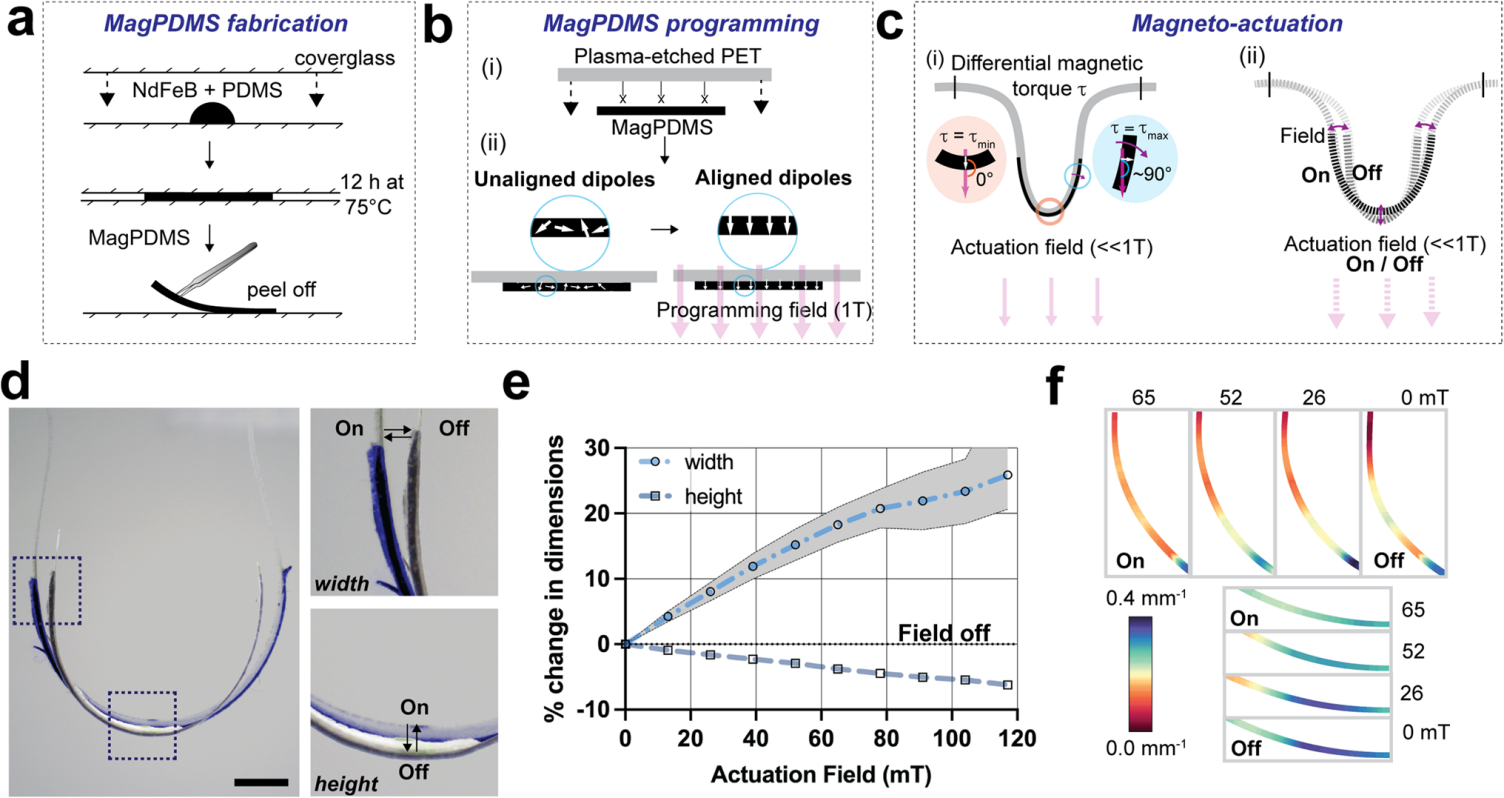
Magneto-active hammock design and characterization. **a** Schematic showing the fabrication of magnetic particle (NdFeB) loaded PDMS (MagPDMS): A droplet of NdFeB loaded (17% volume fraction) PDMS solution is pressed between two Sigmacote-treated coverslips, followed by curing at 75 °C for 12 h and removal from the glass coverslips. **b** Schematic showing the attachment and magnetic programming of the MagPDMS film onto PET membrane: (i) The flat PET membrane is plasma-etched to attach the MagPDMS film, (ii) followed by exposure to a high-intensity programming magnetic field (1 T) to align the magnetic dipoles of the NdFeB particles with the direction of the field. **c** Schematic showing magnetic actuation of the PET-MagPDMS hammock: (i) Upon attachment to the hammock holder, the PET-MagPDMS hammocks are exposed to a low intensity actuation field (<< 1 T) that imposes differential magnetic torque at the bottom and along the arms of the hammock leading to a (ii) net outwards movement of the arms of the hammock and a net upwards movement of the bottom region of the hammock. **d** Representative images of a magneto-active hammock with (On, blue) and without a magnetic field (Off, black) (scale bar 2 mm), showing deformation commensurate with the magneto-generated stresses. **e** Quantification of changes in the dimensions of the hammock at the side (width) and bottom (height) regions in response to increasing actuation field intensities.** f** Curvature maps of magneto-active hammock at increasing applied magnetic strength

Given that SAECs established a monolayer at varying curvatures of the PET hammocks, we next focused on recapitulating the dynamic mechanical stress-strain distribution that cells experience within the distal airways in vivo. To achieve this, we first modified a commonly available silicone elastomer (PDMS) with ferromagnetic (NdFeB) particles to obtain elastic MagPDMS films (Fig. 4a) [20, 21]. Next, MagPDMS films were attached to the plasma-etched PET membranes and a high intensity (1 Tesla (T)) magnetic field (programming field) used to rotate and align the magnetic dipoles in the direction of the programming field (Fig. 4b). Upon attachment to the hammock holder, the magnetic dipoles of the NdFeB particles are pointing radially away from the PET membrane. Thus, exposure to a low intensity actuation field (<< 1 T) induces differential magnetic torque that leads to an outward movement of the sides and consequential upward movement of the bottom region (Supplementary Video 1). This movement is explained by the differential interactions of the field with the magnetic dipoles of the NdFeB particles. As the magnetic torque is a function of the sine of the angle between the magnetic field- and dipole- directions, the resultant torque depends on the position of the particles along the hammock (Fig. 4c). Based on these expected movements of the PET-MagPDMS membrane, we next visualized the shape changes of magnetoactive hammocks in response to an applied magnetic field. Changes in shape along (i) the hammock arms originated from the lateral displacement in width and (ii) along the bottom resulted from the movement of the sides (Fig. 4d). Quantification of the displacement showed a 30% increase along the horizontal (width) and a 5% decrease along the vertical (height) directions of the PET-MagPDMS hammock which was a function of the applied actuation field (Fig. 4e), and completely reversible across multiple cycles of actuation for up to 7 days (Supplementary Fig. 5b). Further, the frequency of actuation may be modulated between 1 and 300 cycles/min which maintains reproducible changes in dimensions (Supplementary Fig. 6). Given that these changes in dimensions may govern variations in curvature distribution, we next used ImageJ to map local curvatures as a function of the applied magnetic field strength. Curvature maps showed an overall greater change in curvature along the arms of the PET-MagPDMS hammock when compared to the bottom (Fig. 4f, Supplementary Fig. 7). These measurements align with the observed changes in dimensions (Fig 4d) and the maximum stress and strain values from finite element analysis (Supplementary Fig. 8). Taken together, we engineered a hammock system that enables dynamic changes in local curvature and strain distributions and thus recreates aspects of the expansion and contraction movement of the distal airways that may be modified for higher throughput.

### Dynamic Actuation Increases SAEC Proliferation and Cytoskeletal Strength

**Fig. 5:**
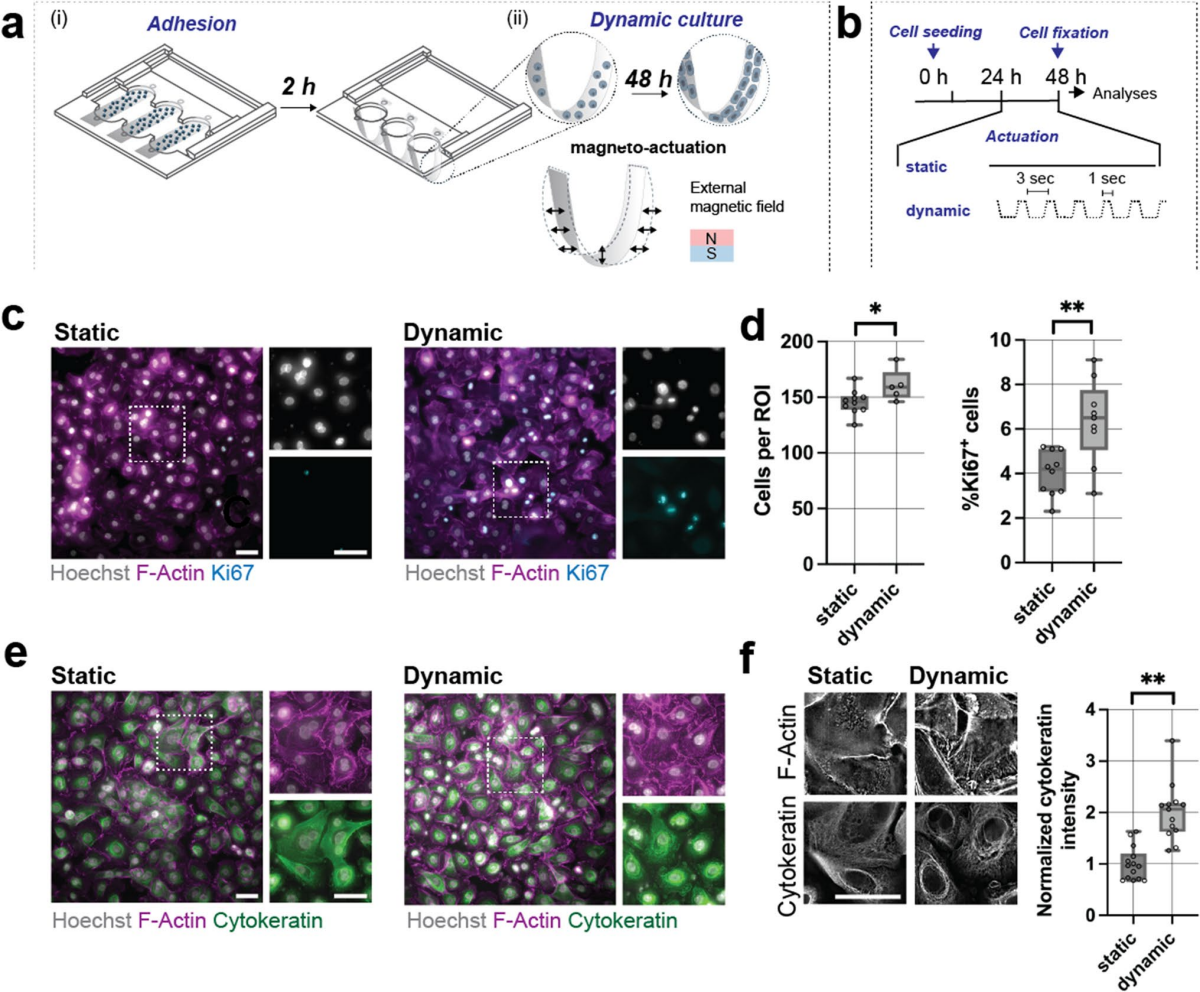
Culture of small airway epithelial cells on dynamically actuated hammocks. **a** Schematic illustrating the small airway epithelial cell (SAEC) seeding and culture protocol: (i) Single cells were seeded on top of the PET membranes and left for 2 h to settle and adhere to the substrate, followed by sliding the holder to form hammocks within each well. (ii) Cells were then cultured for 48 h either under static conditions (magnet off) or dynamic mechanical stimulation (magnet on/off) for 24 h before analysis. **b** Timeline of magnetic actuation protocol of cultured SAECs (static or dynamic culture with 4 s/cycle (15 cycles per min) for 24 h. **c** Representative images of F-Actin and the proliferation marker Ki67 in SAECs cultured under static and dynamic actuation conditions (F-actin (magenta), Ki67 (cyan), Hoechst (grey)), at 48 h in culture (scale bars 50 µm). **d** Quantification of cell density (cells per ROI) and proliferation (Ki67 positive cells) of SAECs at 48 h in culture (*p < 0.05, **p < 0.01 by Student’s *t*-test). **e** Representative images of F-Actin and cytokeratin in SAECs cultured under static and dynamic actuation conditions (F-actin (magenta), cytokeratin (green), Hoechst (grey)), at 48 h in culture (scale bars 50 µm). **f** Representative images and quantification of F-Actin and cytokeratin expression in SAECs cultured under static and dynamic actuation conditions at 48 h in culture (scale bar 50 µm, **p < 0.01 by Student’s *t*-test)

After demonstrating that magnetic hammocks enable reversible expansion and contraction, we next sought to investigate their potential in exerting dynamic forces onto SAECs. Upon seeding cells at a density of 40 × 10^3^ per cm^2^ atop flat PET/MagPDMS membranes, hammock constructs were cultured for an initial 24 h prior magneto-actuation for an additional 24 h (Fig. 5a). The dynamic actuation protocol of 15 cycles per minute (3 s off and 1 s on) was selected to mimic the physiological human breathing pattern [30] and compared to static (non-actuated, magnet off) controls (Fig. 5b). At 48 h of culture, F-Actin staining showed SAEC monolayer formation under both static and dynamic conditions, whereas the number of ki67 positive cells increased under dynamic conditions, indicating increased proliferation in response to changes in curvature (Supplementary Fig. 9) due to dynamic mechanical stimulation (Fig. 5c). In fact, quantification of cell density and Ki67 positive cells showed a nearly 1.5-fold increase for SAECs cultured atop dynamically actuated hammocks when compared to static controls (Fig. 5d). These findings suggest that the magneto-actuation of PET hammocks provides the mechanical signals that enhance the proliferative activity of SAECs. Analysis of bottom and sides of the hammocks showed no difference in cell density and number of Ki67 positive cells, suggesting that the changes in mechano-actuation acts across the entire hammock (Supplementary Fig. 9). Dynamic mechanical stimulation of SAECs on flat PET membranes had no influence on cell function (Supplementary Fig. 10) indicating that the magnetic actuation field itself (65 mT) does not induce cell proliferation. Given that SAECs are directly adhering to the PET membrane and thus are sensing changes in curvature during dynamic mechanical stimulation, we next tested the hypothesis that these changes strengthen the actin cytoskeleton. To test this hypothesis, we stained for actin filaments (F-Actin) using phalloidin and for intermediate filaments using a pan-cytokeratin marker. As expected, both F-Actin and cytokeratin formed a strong cytoskeletal network throughout the SAECs in both static and dynamic conditions (Fig. 5e), which was visible across the monolayers (Supplementary Fig. 10). Interestingly, higher resolution images and quantification of single cells within the dynamically actuated SAEC monolayer showed stronger cytokeratin filaments with a 2-fold increase in overall intensity when compared to static SAECs culture (Fig. 5f). These findings suggest that dynamic mechanical stimulation further enhances proliferation and the strength of the SAEC cytoskeletal network.

## Conclusion

We designed an easy-to-make and easy-to-operate hammock-shaped platform for culture of epithelial cells either under static conditions or dynamic mechanical stimulation. This system is also generalizable to other cell types and applications. Several studies have introduced the application of stretching devices [3, 9] or pneumatic pumps [13] to induce cyclic tensile forces onto lung epithelial cells; however, combining the curvature of the distal airways with tensile forces has remained challenging or requires relatively complex engineering approaches such as microfluidic devices [6] or 3D bioprinting [19]. Here, building upon our previous work on magneto-actuated biomaterials [20], we designed a PET-based hammock-shaped platform that allows for the modeling of curvature and induction of dynamic tensile forces onto the PET membrane. This system is easily accessible for non-engineers and does not require sophisticated tools to assemble the hammock-shaped platform. The reversibility of the system further provides a dynamic cell culture platform to control curvature and tensile strains on demand for controlling cell function, including their differentiation and cell signaling such as the release of cytokines [11, 31, 32]. Additionally, higher throughput of our system will be an important consideration for cell culture. This may be achieved by an array of electromagnets simultaneously actuating multiple samples through strategic positioning of magnetic fields to secure minimum interference [29]*.* In this study, cells were seeded atop the PET membrane to expose them directly to the curvature and strain induced by the hammock and magnetic actuation. However, it is well appreciated that the mechanical properties of the distal airway are much softer than the PET membrane [18], limiting studies of mechanosensing and -transduction. To address this, the addition of customizable hydrogel layers atop the PET-MagPDMS membranes may be used to tune the viscoelastic properties of the hammocks [7, 33–35]. It is also important to note that the size of alveoli ranges between human and animal species [26–28]. While one limitation of the current hammock design is that curvatures are larger than those observed in human lungs, our hammock design is easily tunable to accommodate these different sizes and can be engineered to fit into smaller wells such as 384 well plates. In addition, although this current study focuses on commercially available SAECs, the system is amenable to other cell types, including freshly isolated primary patient-derived cells [36] or human lung cells from induced pluripotent stem cells (iPSCs) [37–39]. The expansion of SAECs in tissue culture plastic and potential induced mechanical memory [40–42] present another limitation of the current study. We chose these cells as an accessible source to mimic the small airway epithelium and because they have been shown to respond to mechanical forces [43, 44]. Other cell types and in particular freshly isolated alveolar epithelial cells that have not been primed on tissue culture plastic, may be used towards studies in alveolar mechanosensing and -transduction. While the current hammock design may not be able to isolate the potential contributions of dynamic fluid flow induced by magnetic actuation, note that in a living system curvature and fluid flow fluctuations occur simultaneously, too. Here, additional studies measuring changes in local fluid flow of magneto-actuated hammocks are necessary to compare the effect of flow patterns with and without magnetic stimulation. This may also motivate future studies to probe the cellular responses to the complex local mechanics of the alveolar walls including multiple and different modes of curvature that epithelial cells likely experience [45, 46]. In summary, we believe that this hammock culture system provides an accessible and versatile strategy to study the combined effect of curvature and strain that is amenable to other tissues (e.g., intestine [47, 48], arteries/veins [49] and upper airways [20, 47]), and can be extended to include the influence of different modes of mechanical activation such as fluid flow and air-liquid interface [43, 50, 51].

## Supplementary Information

Below is the link to the electronic supplementary material.

Supplementary file1 (DOCX 7587 KB)

Supplementary file2 (MP4 1050 KB)

## Data Availability

All data are available upon request from the corresponding author.
