# Supplementary material for: Magnetoactive, Kirigami-Inspired Hammocks to Probe Lung Epithelial Cell Function
Source: Cell Mol Bioeng. 2024 Jul 8;17(5):317–27. doi: 10.1007/s12195-024-00808-z (PMC11538102; doi:10.1007/s12195-024-00808-z)
Supplement: Supplementary file 1 — Supplementary file1 (DOCX 7587 KB) [file 12195_2024_808_MOESM1_ESM.docx]

**Magnetoactive hammocks to probe lung epithelial cell function**

Katherine Wei^1,2,*^, Avinava Roy^1,*^, Sonia Ejike^2^, Madeline K Eiken^3^, Eleanor M Plaster^3^, Alan Shi^1^, Max Shtein^1^, Claudia Loebel^1,2,3,#^

^1^Materials Science & Engineering, College of Engineering, University of Michigan

^2^School of Dentistry, University of Michigan

^3^ Biomedical Engineering, College of Engineering, University of Michigan

^*^These authors contributed equally

^#^Corresponding author: [loebelcl@umich.edu](mailto:loebelcl@umich.edu), ORCID: 0000-0002-3140-5663

**
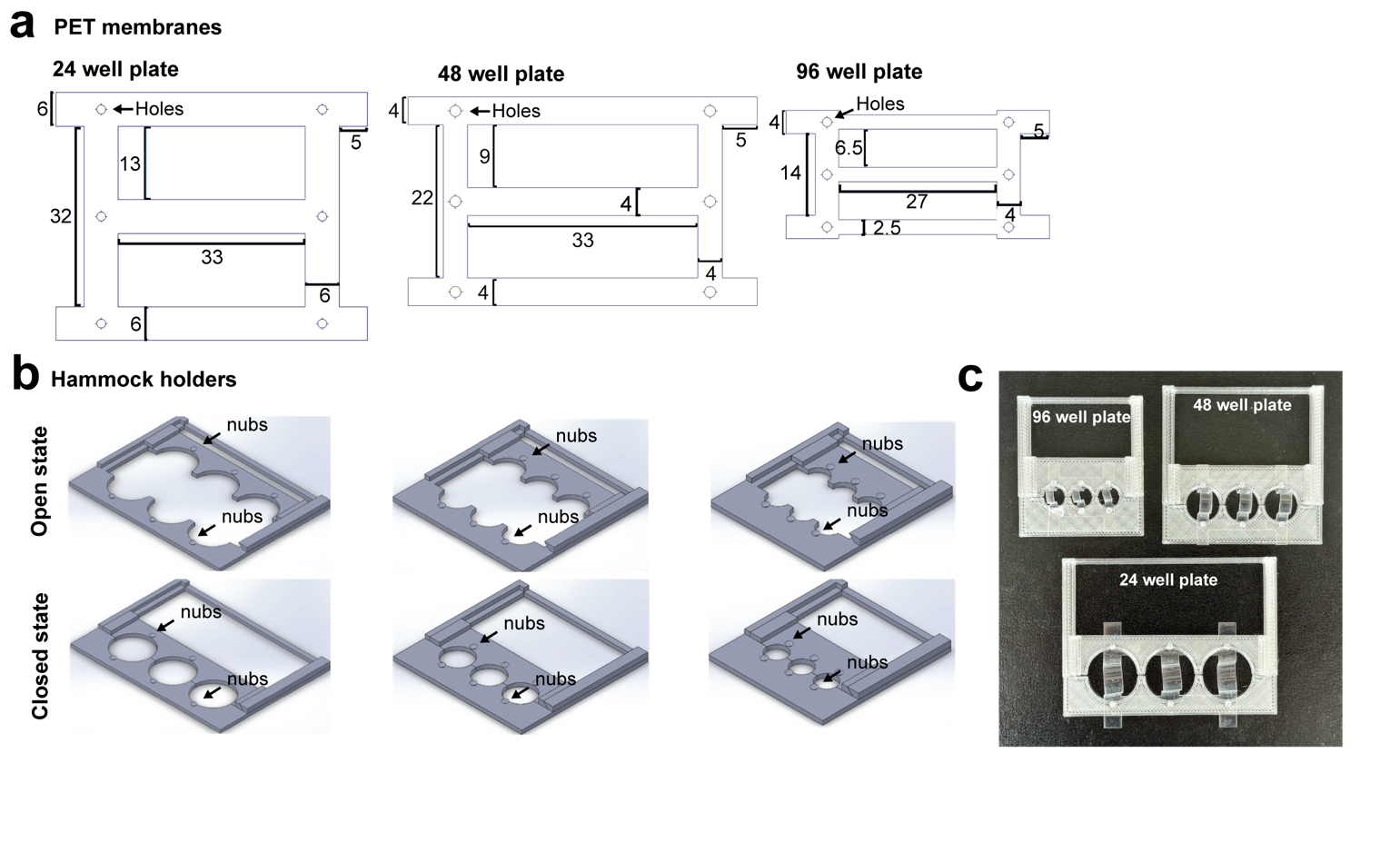
**

**Supplementary Fig. 1** Hammock design for various well plates

**a** Dimensions (in mm) for the PET membranes that fit into 24, 48 and 96 well plates. The membranes feature circular holes that attach to nubs on the holder. The holes have four cuts along the perimeter that are 90˚ from each other. These holes aid in pushing the substrate onto the holder. Additionally, there are four tabs at each corner of the PET substrate. These tabs are points to hold the substrate while mounting it on the holder. **b** Solid work pictures of 3D printed hammock holders that fit into 24, 48 and 96 well plates. **c** Representative photograph of hammocks in hammock holders designed for 24, 48 and 96 well plates.


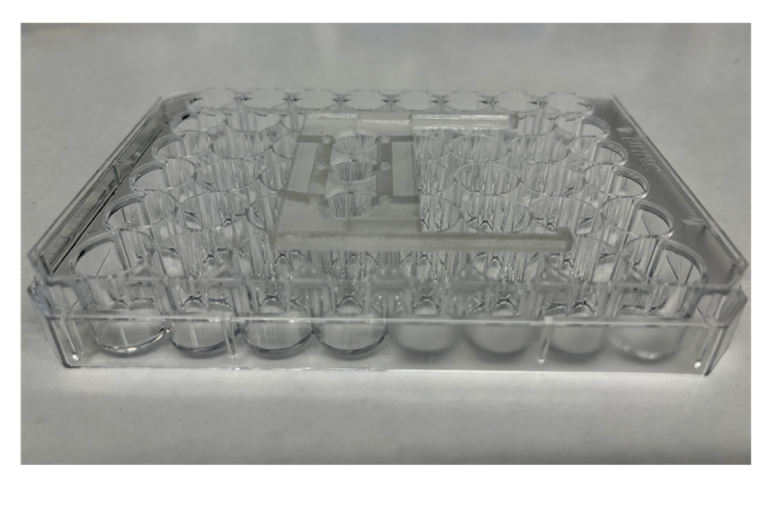

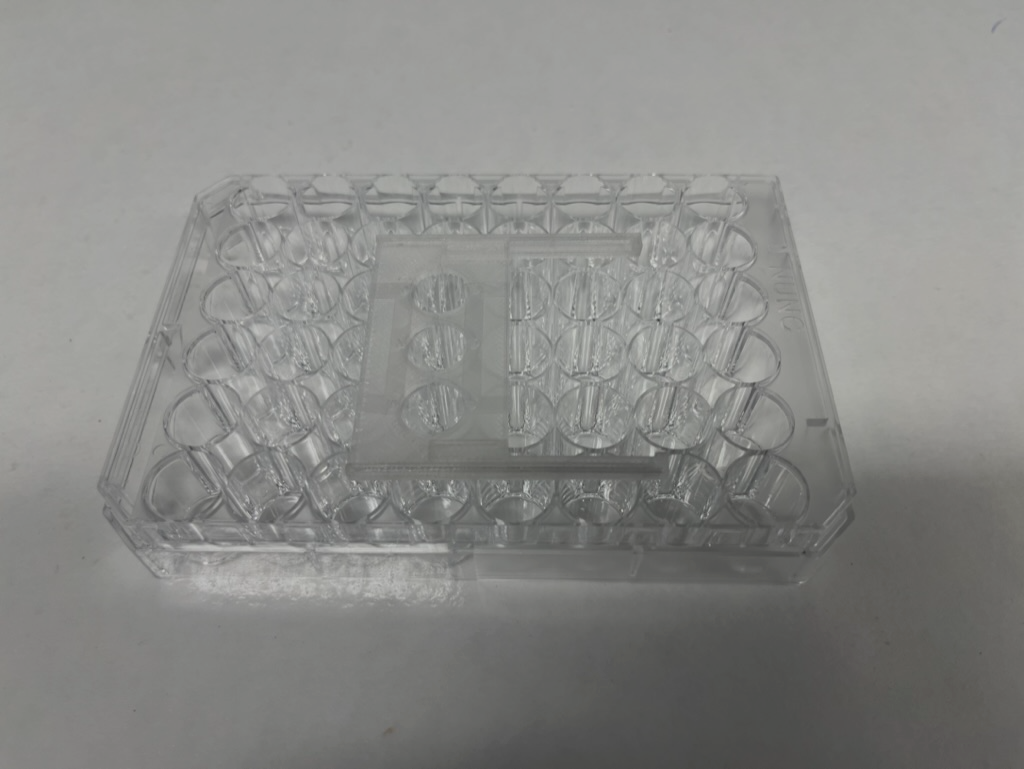


**Supplementary Fig. 2** Hammock cell culture device

Representative photographs of a hammock holder within a 48 well plate for subsequent cell culture (scale bar 5 mm).

**
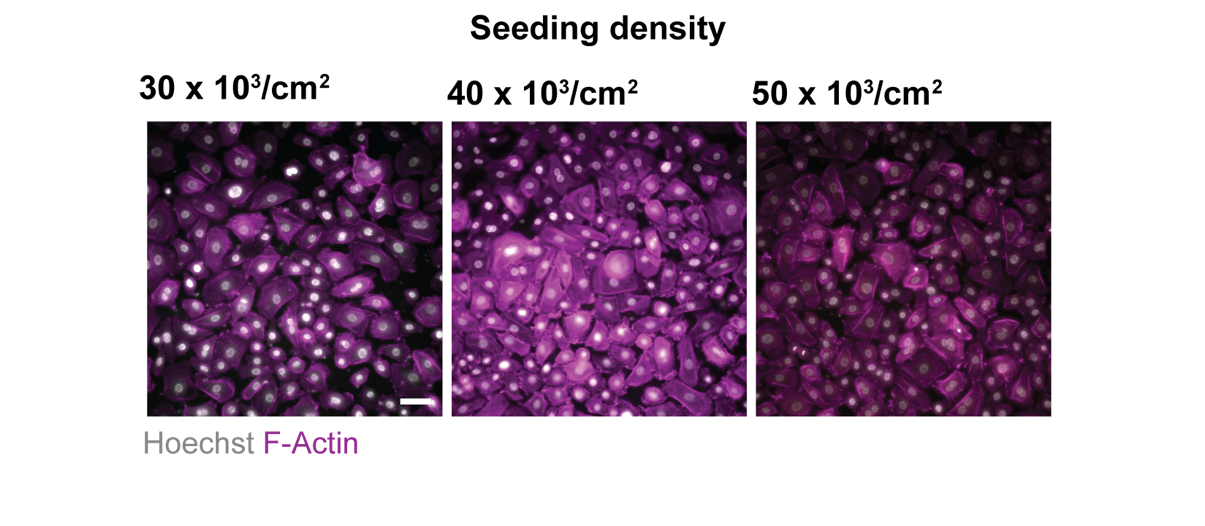
**

**Supplementary Fig. 3** SAEC seeding density on static hammocks

Representative images of small airway epithelial cells (SAECs, F-actin (magenta), Hoechst (grey)) seeded at 30 x 10^3^, 40 x 10^3^, and 50 x 10^3^ cells/cm^2^ at 48 hours in culture (scale bar 50 µm).


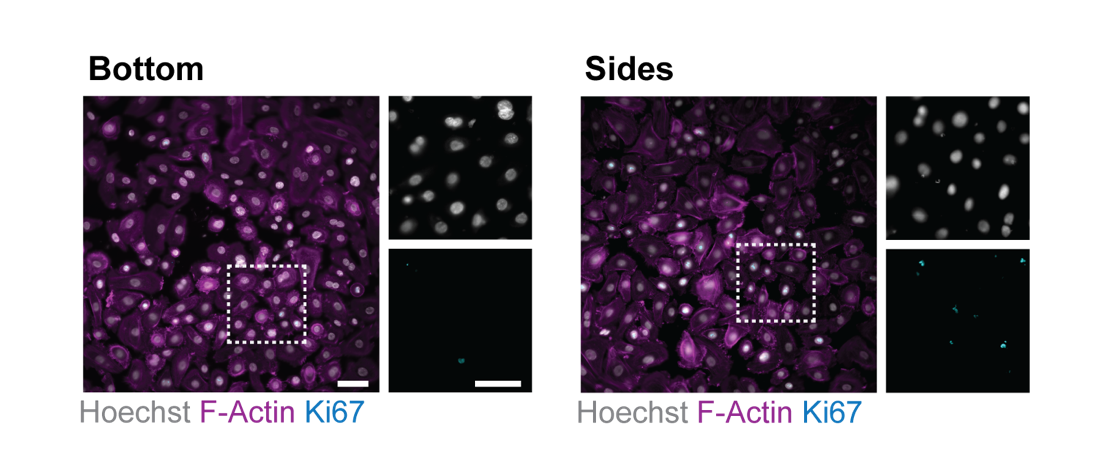


**Supplementary Fig. 4** SAEC proliferation at various locations of static hammocks

Representative images of small airway epithelial cells (SAECs, F-actin (magenta), Hoechst (grey)) at the bottom and sides of static hammocks at 48 hours in culture (scale bar 50 µm).


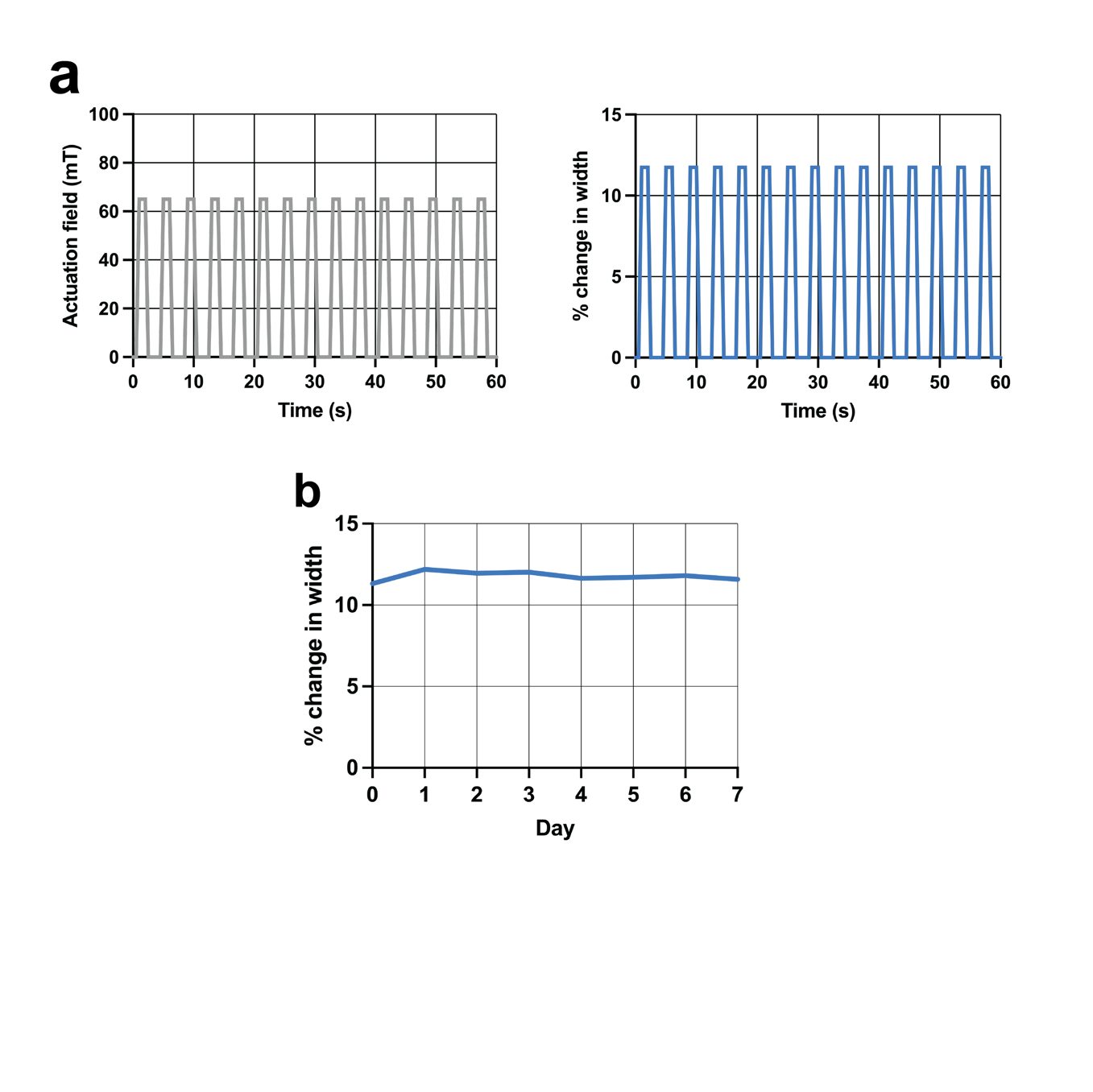


**Supplementary Fig. 5** Short-term and long-term stability of dynamic actuation of magnetic hammock

**a** Quantification of actuation field (left) and percent (%) of change in the width (right) of the hammock. **b** Magneto-active hammock maintains similar change in dimensions across 7 days of continuous actuation (n = 1 representative analysis, 15 cycles/min).

**
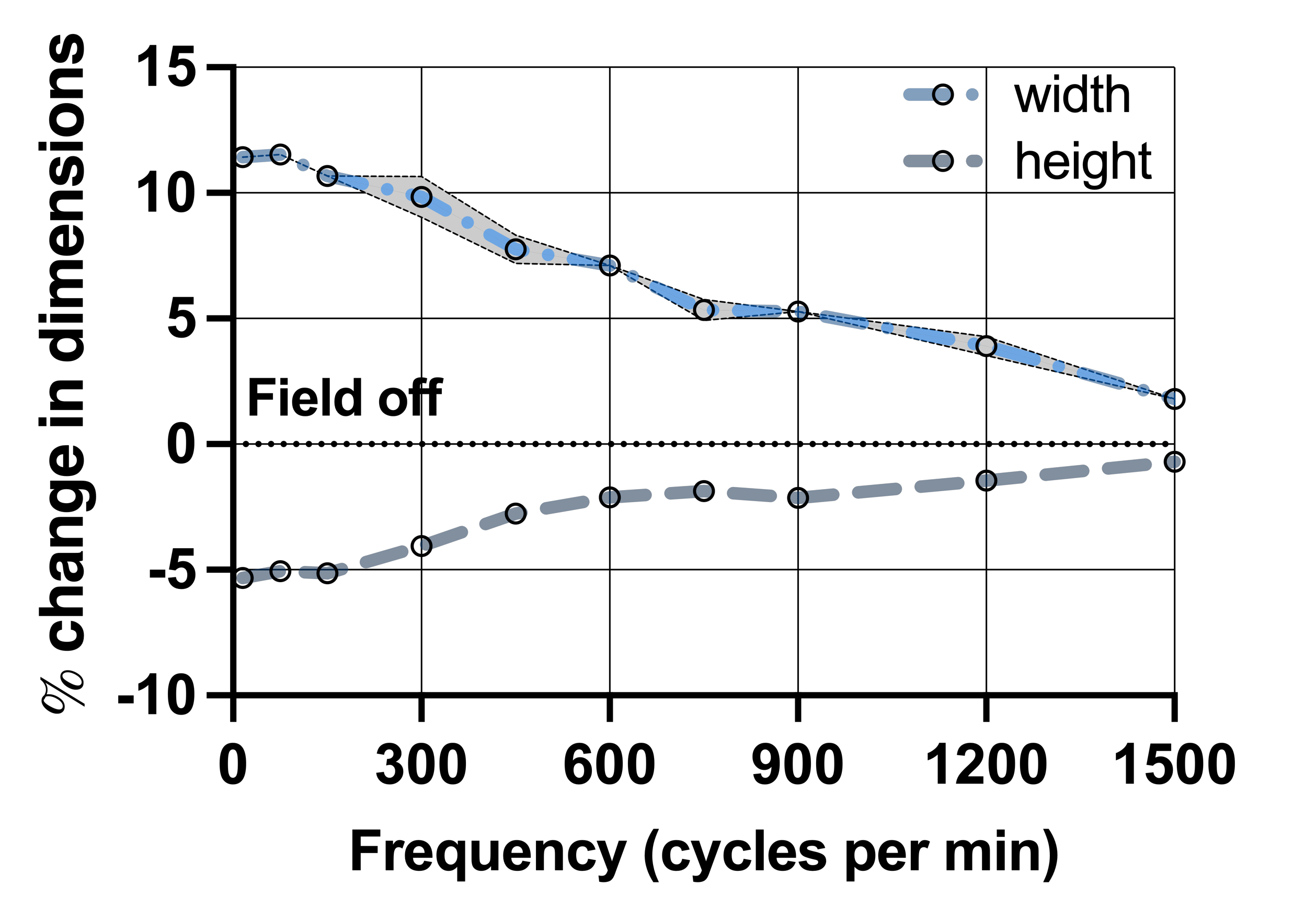
**

**Supplementary Fig. 6** Dimensional changes of hammock as a function of frequency of magnetic field changes.

**
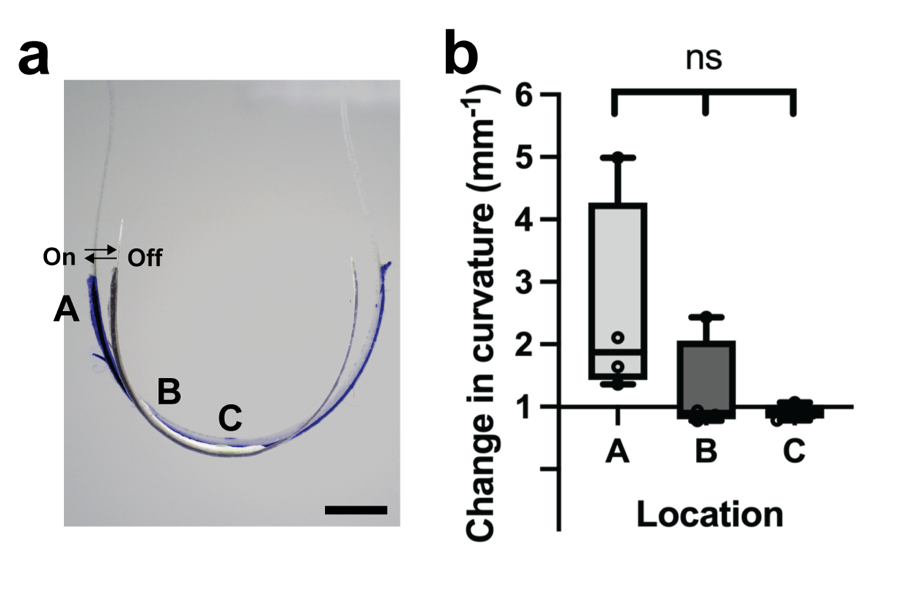
**

**Supplementary Fig. 7** Analysis of Local changes in curvature as a result of magnetic actuation. (a) Representative image showing locations chosen to compute (b) change in curvature at field on (65mT) condition normalized to field off condition (0mT).


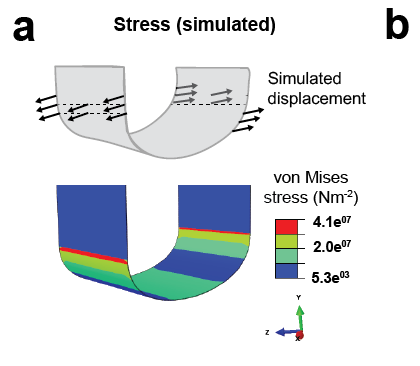


**Supplementary Fig. 8** Maximum stress and logarithmic strain values from finite element analysis simulation. **a** Simulation highlighting emergence of local stress regions along the side and bottom of the magneto-active hammock in response to a low intensity actuation field. The legend shows the von Mises stress distribution. **b** Table showing strain values for three hammocks with horizontal displacements of 0.63, 0.92, and 1.05 mm, respectively, as measured under 65 mT. Strains were identified at specific nodes in the center of the areas of high stress on the arms and bottom of the hammocks. Due to the isotropy of PET, the maximum strain was identified to be the strain of the greatest magnitude.


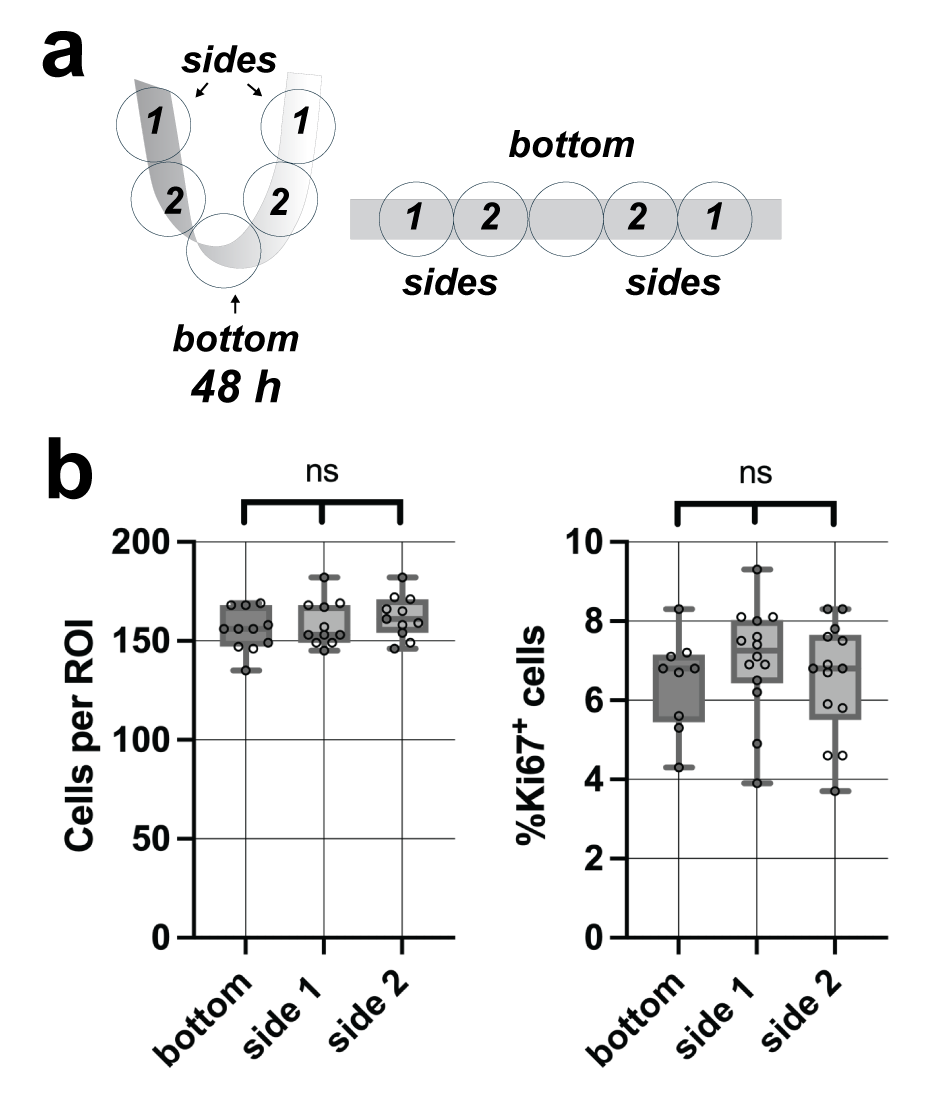


**Supplementary Fig. 9** **a** Schematic of locations across the hammock for quantification of local differences in cell function. **b** Quantification of local differences in cell density (cells per ROI) and proliferation (Ki67 positive cells per total cell number at the bottom and upper (1) and lower (2) sides of the hammocks at day 3 (ns = not significantly different by AVONA and Bonferroni’s multiple comparisons test).


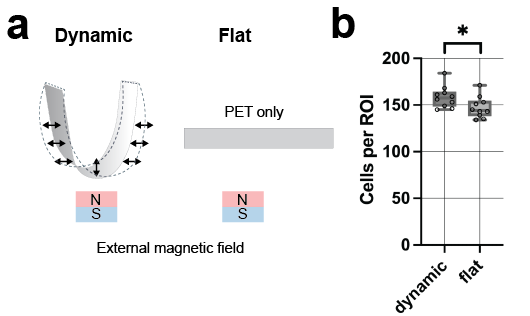


**Supplementary Fig. 10** **a** Schematic illustrating the experimental set-up for culturing cells on magPDMS hammocks with magnetic actuation (dynamic) and PET controls with magnetic actuation (flat). **b** Quantification of cell density (cells per ROI) on magneto-actuated hammocks and flat PET controls at day 3 (p = as indicated by Student's *t*-test).


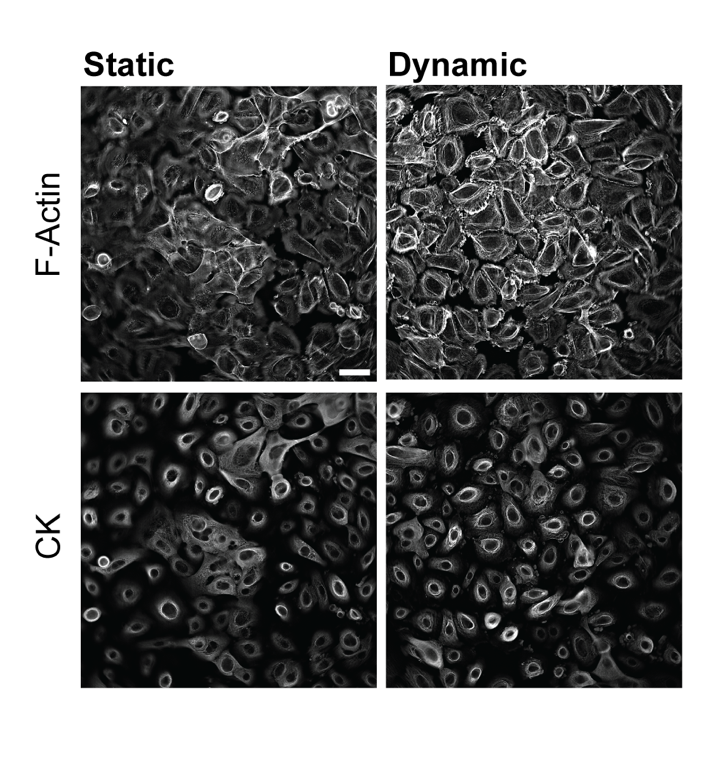


**Supplementary Fig. 11** Cytoskeleton of SAECs in static conditions and dynamic actuation

Representative images of F-Actin and cytokeratin (CK) expression in SAECs cultured under static conditions and dynamic actuation at 48 hours in culture (scale bars 50 µm).
